# Analgesia without sedatives during colonoscopies: worth considering?

**DOI:** 10.1007/s10151-012-0834-5

**Published:** 2012-06-06

**Authors:** S. Eberl, B. Preckel, P. Fockens, M. W. Hollmann

**Affiliations:** 1 Department of Anesthesiology, Academic Medical Center, University of Amsterdam, Meibergdreef 9, 1100 DD Amsterdam, The Netherlands; 2 Department of Gastroenterology and Hepatology, Academic Medical Center, University of Amsterdam, Amsterdam, The Netherlands

**Keywords:** Colonoscopy, Moderate sedation, Alfentanil, N_2_O

## Abstract

Colonoscopy is a proven method for bowel cancer screening and is often experienced as a painful procedure. Today, there are two main strategies to facilitate colonoscopy. First, deep sedation results in satisfied patients but increases sedation-associated risks and raises costs for healthcare providers. Second, there is the advocacy for colonoscopies without any form of sedation. This might be an option for a special group of patients, but does not hold true for everybody. Following Moerman’s hypothesis: “If pain is the crucial point, why do we need sedation?” this review shows the analgesic options for a painless procedure, increasing success rates without increasing risk of sedation. There are two agents, with the potential to be a nearly ideal analgesic agent for colonoscopy: alfentanil and nitrous oxide (N_2_O). Administration of either substance causes the patient to be comfortable yet alert and facilitates a short turnover. Advantages of these drugs include rapid onset and offset of action, analgesic and anxiolytic effects, ease of titration to desired level, rapid recovery, and an excellent safety profile.

## Introduction

Screening by colonoscopy is a proven instrument for early diagnosis of colorectal cancer. This is an important reason why colonoscopies belong to the most frequently performed endoscopic procedures. In the Netherlands, there was a 64 % increase in colonoscopies from 2004 to 2009 [1]. However, motivating patients to participate in colonoscopy screening continues to be a challenge.

The lack of knowledge among patients about the nature of colonoscopy may be an important barrier hindering patients from accepting and undergoing such a screening procedure. Commonly colonoscopy is associated with anxiety and pain. Additionally, patients complain about disruption of normal daily activities by bowel preparation, hangover effects from sedation [2], and need for an escort after the procedure [3]. Dominitz et al. [4] stated that 25 % of patients, who never had a colonoscopy before, were willing to sacrifice, on average, 90 days of their life to avoid the screening procedure. However, after they had a colonoscopy, this number decreased to almost 0 days.

### Pain and discomfort during colonoscopy

Pain during colonoscopy is considered to be visceral, resulting from the activation of sensory afferent nerves that innervate the intestines. The main factors involved include stretching of the sigmoid wall and mesenteric attachments from looping of the colonoscope shaft and overinsufflation [5]. Visceral pain often triggers autonomic responses, for example, sweating, bradycardia, dizziness, hypotension, and nausea.

Although pain is a physiologic response to tissue damage, it also includes emotional and behavioral responses based on individuals’ past experiences and cultural background, which often seem to be resistant to analgesic treatments. Pain is less well tolerated by younger females with a low body mass index (BMI) and is better accepted by older patients. Unfortunately, it is impossible to predict how painful the examination for the individual patient will be.

### Sedation for colonoscopy

Sedation during colonoscopy is currently a subject of debate; in the United States (US), sedation has become standard for colonoscopies, and different studies advocate either moderate or deep sedation. Other parts of the world argue for medication-free colonoscopy [6–10]. In one US study, only 16.9 % of 434 patients were willing to undergo colonoscopy without sedation [11]. However, another study reported that in 23 % of patients, unsedated colonoscopy could be performed with excellent patient satisfaction and an acceptable comfort level [12]. Eckardt et al. [7] showed, in a study on 2,500 patients, that 95 % of all patients could undergo colonoscopy without sedation when experienced colonoscopists and optimal equipment were present. Unfortunately, the authors did not report data on patient satisfaction. Nowadays, use of new colonoscopes [13], the water method [14], and experienced endoscopists make colonoscopy without sedation possible for a motivated group of patients. This finding is supported by Rex et al. [15]. Success rates depend on appropriate patient selection [10]. Male gender, higher levels of education, low preprocedural anxiety, and a personal preference for procedures without sedation are predictors of a successful sedation-free procedure [11]. However, unpredictable individual anatomic variations can result in inacceptable discomfort for the patient and worse procedural conditions for the gastroenterologist. Baudet et al. [16] reported increased complication rates during colonoscopy without the use of sedation (57 vs. 22 %; *P* < 0.001).

### Modes of analgosedation

Sedation guidelines have universally defined levels of sedation, reaching from moderate to deep sedation.

*Deep sedation* is generally achieved using propofol, which has a rapid onset and short duration of action, allowing for a reduced recovery time. Therefore, there is increasing interest in propofol sedation among gastroenterologists. However, propofol has a relatively narrow therapeutic range that enhances the risk of sedation-related cardiopulmonary events. Most states in the United States do not allow the use of propofol by non-anesthesiologists. The European guidelines concede the administration of propofol to trained nurses or endoscopists who are solely responsible for sedation [17]. However, this permission only concerns moderate, but not deep sedation.

This means deep sedation is likely to be more resource-intensive due to a higher need for specialized staff and monitoring [18]. The percentage of colonoscopies performed with the participation of anesthesia professionals is expected to rise from 23.9 % in 2007 to 53.4 % by 2015, respectively [19]. In view of this dramatic increase, health insurance companies are attempting to restrict coverage for anesthesia professional-delivered sedation [18].

Deeply sedated patients may have inadequate spontaneous ventilation and therefore may require assistance to maintain a patent airway. Closed claim analyses of anesthesia suggest that serious injury can occur during deep sedation, even with properly trained providers [20]. Coté et al. [21] found a percentage of 12.5 % sedation-related hypoxemic events during propofol sedations performed by anesthesia nurses. In a review of over 20,000 reports in the Clinical Outcomes Research Initiative Database, sedation-related complications occurred in 1.3 % [22]. The most common complications were respiratory depression (0.75 %) and cardiovascular events (0.49 %), delayed recovery of psychomotoric function, and delayed discharge. Furthermore, deeply sedated patients are not able to change position from lateral decubitus to supine without assistance, which makes it difficult to maneuver the patient.

*Moderate sedation* was defined as a drug-induced depression of consciousness in which patients could purposefully respond to verbal commands, and where spontaneous ventilation was adequate, without the risk of losing a patent airway [23]. The drugs most commonly used for moderate sedation are midazolam (47 %), other benzodiazepines (4 %), spasmolytics (11 %), and other drugs (5 %), mostly combined with an analgesic, for example opioids (33 %) [24]. A combination of two or more analgosedatives was used in 37 % of the procedures performed. This combination provides excellent analgosedation during colonoscopy, but increases the risk of more deep sedation and more frequent respiratory depression.

The duration of action of the respective drugs might last longer than the duration of the procedure, resulting in prolonged recovery with a delay in hospital discharge, increased costs, and disruption of daily activities of the patients.

### The ideal agent

The properties of an ideal analgesic agent for colonoscopy aiming at a comfortable yet alert patient and facilitating a rapid turnover of patients would include rapid onset and offset, analgesic and anxiolytic effects, ease of titration to a desired level, rapid recovery, and an excellent safety profile with the existence of a specific, rapidly acting antagonist—all this without the need for additional personnel.

Following the hypothesis “If pain relief is adequate during colonoscopy, sedation is no longer being required” [25], the question arises whether analgosedation could be achieved using analgesics alone. Various studies on sedation regimens have been published, but only a few concentrated solely on analgesic agents [26–28].

### Meperidine

Meperidine is a synthetic analgesic, which has its peak onset of action within 10–15 min and then lasts for 2 h with a plasma half-life of 3–4 h. It is rapidly metabolized to normeperidine, which undergoes renal excretion with an elimination half-life of 17 h. The pharmacokinetic profile strongly argues against the use of meperidine for relatively short procedures like colonoscopies [29].

### Fentanyl

Fentanyl is an opioid that has a faster recovery profile than meperidine. Onset of action is within about 1–2 min, peak effect occurs at 3–5 min, and duration of action ranges between 30 and 60 min.

For colonoscopies, fentanyl is usually combined with a benzodiazepine or propofol. Only Lazaraki et al. [26] evaluated the efficacy and safety of fentanyl alone (<0.5 μg/kg, mean 36 μg) in comparison with midazolam (2 mg, mean 4.6 mg). Fentanyl provided more rapid recovery than midazolam, combined with lower mean discomfort (0.4 vs. 1.0) and pain scores (2.59 vs. 4.43). No adverse events occurred in the fentanyl group, whereas in the benzodiazepine group, a decrease in oxygen saturation was noted in 35 % of the patients.

### Remifentanil

Remifentanil is an ultra short-acting synthetic opioid (onset 30–60 s, peak effect after 2.5 min) with an analgesic potency similar to that of fentanyl, and is metabolized by nonspecific esterases. Owing to remifentanil’s rapid systemic elimination, with a half-life of 8–10 min, it should have pharmacokinetic advantages in clinical situations requiring predictable termination of effect. Akcaboy et al. [30] showed that low-dose remifentanil (0.05 μg/kg/min) continuous and bolus injection—in combination with 2 mg midazolam—can provide adequate sedation, amnesia, and better analgesia with lower discomfort scores than propofol infusion during colonoscopy. However, remifentanil induced nausea and vomiting during the recovery phase and delayed patients’ discharge. Hemodynamic instability, consisting of a significant drop in blood pressure and significant bradycardia, and impaired oxygen saturation levels were additional disadvantages of remifentanil bolus injection. Nonetheless, gastroenterologist and patient satisfaction was higher, and duration of colonoscopy was shorter compared with the propofol group. This could be explained by better cooperation of the patients. Similar results were reported by Fanti et al. [31] using remifentanil patient-controlled analgesia (PCA) (0.5 μg/kg) in combination with midazolam.

Moerman et al. [25] compared high-dose remifentanil (0.5 μg/kg followed by 0.2 μg/kg/min) with propofol (1 mg/kg followed by 10 mg/kg/h). Adequate conditions for colonoscopy could be obtained using both drugs. Emergence times and recovery of cognitive function were faster with remifentanil, and hemodynamic disturbances were reduced compared to propofol. Remifentanil-induced respiratory depression was found to be a significant problem in this study. Patient satisfaction was significantly higher in the propofol group than in the remifentanil group, probably due to a deeper level of sedation after use of propofol. Greilich et al. [32] compared remifentanil versus meperidine in older patients undergoing colonoscopy. Although overall satisfaction was the same in both groups, verbal pain and anxiety scores during parts of the procedure were higher in the remifentanil group compared to the meperidine group.

In a recent study by Manolaraki [33], the safety and efficacy of remifentanil (loading dose of 1 μg/kg over 60 s followed by continuous infusion at a rate of 0.05–0.2 μg/kg/min) during colonoscopy were compared with the standard combination of midazolam and pethidine. Although mean levels of pain with remifentanil were higher than those with midazolam and pethidine, there was no difference in patient and endoscopist satisfaction between the two groups. Patients in the remifentanil group experienced significantly less respiratory depression, most likely due to a careful titration of remifentanil to reach the desired sedation level. It is important to note that a much faster discharge of patients in the remifentanil group was observed. The necessity for continuous application and the drug’s negative side effects (nausea, vomiting and possible hemodynamic and respiratory complications) are serious limitations for routine use of remifentanil. Because only trained users (anesthesiologists and anesthesia nurses) would administer remifentanil, additional staffing costs will be associated with this analgesic regimen.

### Alfentanil

Alfentanil is a short-acting μ-opioid analgesic chemically related to fentanyl, but less lipophilic. Comparable to remifentanil, alfentanil has a rapid onset of action. The maximal analgesic and respiratory depressant effect occurs within 1–2 min. Alfentanil is metabolized mainly within the liver, with only 1 % of the active substance found non-metabolized in the urine. Thus, in patients with liver dysfunction, a more prolonged and pronounced effect can be expected. The terminal elimination half-life is 90–111 min. Dose dependency allows for achieving different levels of awareness, cooperation, and psychomotor capacity more easily.

The only study addressing the use of alfentanil (10 μg/kg) for colonoscopies as a mono-drug was performed by Di Palma et al. [27]. The authors compared alfentanil with midazolam/alfentanil (*n* = 11) and meperidine/midazolam (*n* = 11). Patients receiving alfentanil (*n* = 13) were less likely to require oxygen supplementation because of desaturation (8 vs. 55 % with alfentanil/midazolam and 27 % with meperidine/midazolam) and suffered from less pain. There were no differences in tolerance and discomfort, ease in operation, recovery time, complications, electrocardiogram (ECG) changes, and effects on blood pressure, and therefore, the authors concluded that alfentanil alone had no further advantage. However, the safety aspect—significantly less desaturation episodes—makes the substance worth to be examined in more detail.

Usta et al. [34] compared patient-controlled analgesia (PCA) with alfentanil (mean 1,000 μg) versus fentanyl (mean 80 μg) for colonoscopies. Both opioids were given in combination with midazolam (2.34 ± 0.96 mg in the alfentanil group and 2.16 ± 0.9 mg in the fentanyl group). It is worth mentioning that analgesia was not completely patient-controlled. Patients received a loading dose of 500 μg alfentanil and were then asked to request a further bolus (by pushing the button) when they felt pain. If the sedation score exceeded 3 (OAA/S), further midazolam was added. Patients in both groups had the same satisfaction score after colonoscopy and were willing to undergo the procedure again with the same analgesic regimen. No adverse events (e.g., respiratory depression and hemodynamic changes) were observed. As expected, recovery was significantly shorter with the use of alfentanil compared to fentanyl. The authors’ conclusion focused on alfentanil, although midazolam was also administered as a sedative agent.

No other studies addressed the use of alfentanil for colonoscopies. In neurosurgical patients undergoing stereotactic brain biopsy, Bilgin et al. [35] compared the effects of alfentanil, fentanyl, and remifentanil analgosedation combined with midazolam on hemodynamic and respiratory parameters. Alfentanil (10 μg/kg) initially led to a reduction in minute volume and blood oxygen saturation (SpO_2_), though without any clinically relevant respiratory depression. This effect was aggravated by additional sedation using benzodiazepines [36].

### Nitrous oxide (N_2_O)

Nitrous oxide is an inert gas of low solubility which is rapidly absorbed (within 60 s) and eliminated unchanged via the lungs. Available in a fixed 50:50 combination with oxygen (Entonox^®^/Relivopan^®^/Livopan^®^), it has been widely used as an analgesic in obstetric and dental practice for more than 160 years [37]. It has a rapid on and offset, with minimal side effects. The analgesic effect is attributed to the inhibition of N-methyl D-aspartate (NMDA)—receptors—and the anxiolytic and sedative effect to the activation of gamma-aminobutyric acid (GABA)—receptors. In animal studies, N_2_O induced the release of opioid peptides in the brainstem followed by the activation of descending noradrenergic inhibitory pathways. Hence, N_2_O modifies pain processing in the spinal cord and induces analgesia—without loss of consciousness [38, 39].

Welchman et al. [40] performed a systematic review comparing N_2_O to intravenously administered opiates with or without midazolam in patients undergoing colonoscopy. Unfortunately, only a small number of patients were included and great diversity existed among them. In addition, no validated scores were used to assess patient satisfaction [41]. The data showed that N_2_O use on demand was not sufficient to adequately reduce pain, probably because a short lag time exists before analgesia is reached by N_2_O. Løberg et al. [42] showed that N_2_O on demand is not an effective substitute for intravenous medication in patients undergoing colonoscopy. Combining a loading dose of N_2_O for 2 min with self-administration on demand thereafter revealed N_2_O to be superior to standard fentanyl/midazolam analgosedation in terms of pain scores, patient satisfaction, and willingness to undergo the same procedure again using the same sedation regimen [43]. In contrast, Forbes et al. [44] reported that Entonox^®^ was less effective than meperidine/midazolam with respect to pain scores, but allowed for faster recovery. Prediction of painful maneuvers during colonoscopy is difficult, and the patient might use N_2_O too late to achieve an adequate pulmonary concentration necessary for subsequent pain reduction. Maslekar et al. compared continuous inhaled Entonox with patient-maintained target controlled infusion with propofol. They found no differences between N_2_O and propofol regarding pain relief, sedation, and mobility of the patients [45].

N_2_O for short-acting procedures is considered safe [46]. Onody et al. [47] analyzed 35,828 questionnaires and demonstrated an incidence rate of all adverse effects of 4.4 %, 86 % of which were gastrointestinal (nausea, vomiting) and neuropsychiatric (dizziness, headache, and hallucinations) disturbances.

The only proven toxic effect of N_2_O concerns interaction with vitamin B12, which also depends on duration (6 h) and extent of exposure. Animal studies suggest a problem associated with chronic exposure to N_2_O. The exact level of exposure that induces patient harm cannot be predicted. Only long-term exposure to N_2_O in sufficient concentrations seems to produce irreversible, toxic changes and has been associated with reproductive, hematologic, immunologic, neurologic, liver, and kidney disorders. Hence, administration of N_2_O to patients for a short-term colonoscopic procedure seems to be safe. Attention should be paid to the safety of personnel working in environments in which N_2_O is used the whole day, especially without an adequate extraction system.

The safety level for N_2_O exposure is yet not clearly defined. The National Institute for Occupational Safety and Health recommended an exposure limit for N_2_O of 25 parts per million (ppm) as a time-weighted average for a normal 8-h workday and a 40-h workweek [48]. The American Conference of Governmental Industrial Hygienists has assigned N_2_O a threshold limit value of 50 ppm as a time-weighted average. In Germany, the Occupational exposure limit is 100 ppm [49]. Lacking exact data, it is important to minimize exposure.

Every N_2_O apparatus must have a scavenging system [50] with adequate extraction which routinely should be checked for leaks. Furthermore, there must be a reasonable exchange of air in the room with at least 2–3 air exchanges per hour when N_2_O is used. Patients should wear an on-demand valve mask perfectly fitting their faces and be advised not to speak during colonoscopy. After finishing the procedure and stopping N_2_O, patients should receive 100 % oxygen for 3–5 min via the mask.

## Conclusions

Overall, the discussion on the ideal agents demonstrates that in fact none of the agents is ideal compared to standard conscious or deep sedation. Almost all have side effects, have lower patient satisfaction scores, have been used with sedatives, or have been studied in very small trials. But if pain is relieved adequately during colonoscopy, sedation is indeed not required in a very large number of patients. The use of N_2_O instead of intravenous drugs is “no laughing matter” [51], for several reasons: N_2_O with a loading dose and continuous administration provides adequate analgesia with a patient being awake and cooperative. After cessation, the patient is awake, ready to get the information necessary, and to leave the hospital soon after the procedure. In particular, patients who live alone or wish to drive home on their own may benefit from the rapid recovery of psychomotoric function provided by N_2_O. However, there are some limitations of N_2_O like uncertainty about chronic side effects and need for air-conditioning and efficient ventilation together with efficient active scavenging systems.

Alfentanil is a strong analgesic, facilitating a fast turnover of satisfied, pain-free patients, who are able to cooperate with the endoscopist. Its respiratory depressant effects are without clinical impact. Moreover, all actions of alfentanil can be immediately reversed by naloxon, making the substance safe in general use.

Further studies are needed to assess efficiency and last but not least patient and physician satisfaction levels with use of these two forms of analgesia.
